# Correlations between Microstructure and Residual Stress of Nanoscale Depth Profiles for TSV-Cu/TiW/SiO_2_/Si Interfaces after Different Thermal Loading

**DOI:** 10.3390/ma16010449

**Published:** 2023-01-03

**Authors:** Min Zhang, Fangzhou Chen, Fei Qin, Si Chen, Yanwei Dai

**Affiliations:** 1 Institute of Electronics Packaging Technology and Reliability, Faculty of Materials and Manufacturing, Beijing University of Technology, Beijing 100124, China; 2 Science and Technology on Reliability Physics and Application of Electronic Component Laboratory, China Electronic Product Reliability and Environmental Testing Research Institute, Guangzhou 510610, China; 3 Beijing Key Laboratory of Advanced Manufacturing Technology, Faculty of Materials and Manufacturing, Beijing University of Technology, Beijing 100124, China

**Keywords:** residual stress, TSV interconnected interfaces, ion-beam layer removal (ILR) method, microstructure, thermal loading

## Abstract

In this paper, the residual stresses with a nanoscale depth resolution at TSV-Cu/TiW/SiO_2_/Si interfaces under different thermal loadings are characterized using the ion-beam layer removal (ILR) method. Moreover, the correlations of residual stress, microstructure, and the failure modes of the interfaces are discussed. The residual stresses at the interfaces of TSV-Cu/TiW, TiW/SiO_2_, and SiO_2_/Si are in the form of small compressive stress at room temperature, then turn into high-tensile stress after thermal cycling or annealing. In addition, the maximum residual stress inside the TSV-Cu is 478.54 MPa at room temperature, then decreases to 216.75 MPa and 90.45 MPa, respectively, after thermal cycling and annealing. The microstructural analysis indicates that thermal cycling causes an increase in the dislocation density and a decrease in the grain diameter of TSV-Cu. Thus, residual stress accumulates constantly in the TSV-Cu/TiW interface, resulting in the cracking of the interface. Furthermore, annealing leads to the cracking of more interfaces, relieving the residual stress as well as increasing the grain diameter of TSV-Cu. Besides this, the applicability of the ILR method is verified by finite element modeling (FEM). The influence of the geometric errors of the micro-cantilever beam and the damage to the materials introduced by the focused ion beam (FIB) in the experimental results are discussed.

## 1. Introduction

Three-dimensional (3D) packaging based on copper filled through silicon via TSV-Cu technology takes advantage of its characteristics of low power consumption, light weight, and small volume; it has been widely used in MEMS, mobile phones, memory products, CMOS image sensors, and biological application equipment [1]. However, with the development of heterogeneity integration, the layout design of crucial TSV interconnection structures becomes finer. The substantial initial residual stress accumulated during the fabrication process leads to the formation of voids, warpage, and interface cracks of the interconnection structure [2,3,4,5]. Moreover, during the service stage, the finer interconnection structure leads to an increase in power and heat flux density for electronic devices. Thus, larger thermal stresses between the interconnected structures are generated due to the mismatch of the thermal expansion coefficient (CTE), which causes further reliability problems, such as TSV-Cu protrusion, the cracking of solder joints, and interface delamination [6,7,8]. A comprehensive understanding of residual stress could provide significant theoretical guidance for the fabrication of semiconductor devices.

The accumulation of local mechanical stress in TSV interconnection structures leads to stress concentrations, resulting in the failure of devices. Therefore, a characterization method of local stress is urgently needed for the purposes of reliability assessment. Due to the submicron-length scale of the TSV interconnection structure, traditional macro-test methods, such as drilling, slicing, the contour method, the blind hole method, etc., are not available to obtain the residual stress at micro- and nanoscale resolution. The wafer curvature method has generally been adopted to measure the thickness-averaged residual stress of a blanket multilayer structure fabricated by a semiconductor process [8,9,10]. The average residual stress of a TSV-based silicon interposer was also measured using this method [11]. However, the local residual stress of TSV-Cu cannot be obtained using the wafer curvature method. In addition, the characterization of local residual stress on TSV-Cu surface can be accomplished via Raman spectroscopy, X-ray microdiffraction, second-harmonic spectroscopy, and the nanoindentation test [12,13,14,15,16,17,18]. Nevertheless, the above characterization methods have strict requirements regarding the material surface. Moreover, the residual stress can only be measured within a small range on the material surface, and the stress in the depth direction of the interconnection structure is still unmeasurable.

Finite element modeling (FEM) can be used to calculate the local thermal mechanical stress distribution of a TSV interconnection structure under different thermal loadings [19,20,21,22]. For example, the distributions of stress and strain of TSV interconnection structure under thermal cycling from 25 °C to 125 °C were calculated by FEM, and the calculated strain was compared with the strain measured with digital image speckle correlation (DISC) [23]. In addition, the fracture parameters of the TSV-Cu/Si interface were measured and referred to as the input of material parameters in the FEM to analyze the failure possibilities of the interconnected interfaces under various thermal mechanical loadings [19,20]. Although FEM is an efficient method by which to calculate the local stress of interconnection structures, the accuracy of the calculation depends closely on the accurate physical/mechanical property parameters of the various interconnected materials, which are still difficult to acquire. Furthermore, the generation and accumulation of initial residual stress in the fabrication processes are complex physicochemical and mechanical changes, which are difficult to consider comprehensively in FEM. The ignorance of the actual mechanical parameters and the initial stress state in FEM would result in a great difference between the calculated stress and the realistic stress.

The residual stress of GaN/Al_x_Ga_1−x_Si/Si interconnection structure was measured by ion-beam layer removal (ILR) and the curvature method. It was found that the thickness-averaged stress was basically the same with the two methods; however, the curvature method cannot accurately measure the locally concentrated stress [24,25]. The local stress gradient distributions of the Si/W/Cu/W and Si/Cu/W/Cu-interconnected structures were measured by the ILR and X-ray microdiffraction methods, respectively. It was found that the inner stresses in the individual inner materials from the two methods were consistent, but the stresses at the interface, with a range of several grains displayed, showed a pronounced difference due to X-ray microdiffraction [26]. It was verified that the above limitations and shortcomings of measuring the local residual stress can be overcome by the ILR method. The ILR method first proposed by S. Massl was combined with the cantilever theory to measure the residual stress gradient distribution of the Ni/SiOx/SiNx/Si-interconnected structure at nanoscale-depth resolution [27,28]. Based on this method, the residual stress of the W/TiN/Si-interconnected structure was measured and the influence of the shape errors of the micro cantilever, machined by a focused ion beam (FIB), on the experimental results was discussed [29]. As for the same W/TiN/Si structure, the microstructure of the W metal layer was further observed. It was found that the function of grain size and residual stress conformed to the Hall–Patch relationship [30]. However, the correlations between the local residual stress and the microstructure of the interconnected structure have not been fully elaborated in available investigations. Moreover, the interaction mechanisms between the local gradient stress and microstructure under different thermal loadings have not been studied.

The TSV technology is the core of 3D packaging, which is a powerful solution for more than Moore’s Law in the area of integrated circuits. It should be mentioned that TSV-based packaging structures involve multi-materials and multi-interfaces, which function as conductive layers, barrier layers, insulator layers, and substrate layers. For example, TSV-Cu/TiW/SiO_2_/Si interconnected interfaces meet the above functions, and their interfacial integrity is an essential condition to ensure the high reliability of TSV-based 3D packaging devices. However, micro voids and cracks are generally generated at the TSV-Cu/TiW/SiO_2_/Si-interconnected interfaces, due to multiple thermal loadings caused by the manufacturing processes, put TSV-based devices at great risk of reliability issues [20,31,32]. Investigations have confirmed that the local residual stress concentration and microstructure evolutions at TSV-Cu/TiW/SiO_2_/Si interfaces are the main formation mechanisms of micro voids and cracks at the TSV-Cu/Si interfaces [33,34]. Nevertheless, the correlations between local residual stress and the microstructural evolutions of TSV-Cu/TiW/SiO_2_/Si interfaces have not been investigated. Moreover, the combined effects of local residual stress and the microstructural evolution of interfacial failure mechanisms also need to be clarified.

In this paper, the residual stress of the nanoscale depth profiles for the TSV-Cu/TiW/SiO_2_/Si-interconnected structure under different thermal loadings is investigated using the ILR method. The stress gradient and failure modes of the interconnection structure at room temperature, in thermal cycling, and annealing are studied, respectively. The microstructure of TSV-Cu under different thermal loadings is analyzed, and the relationships with the stress gradient and failure modes are established. In addition, the applicability of the ILR method to measuring the residual stress gradient of the TSV interconnection structure is verified by FEM. 

## 2. Preparation of Samples and the ILR Method

### 2.1. Fabrication of a Micro Cantilever Beam

**Figure 1:**
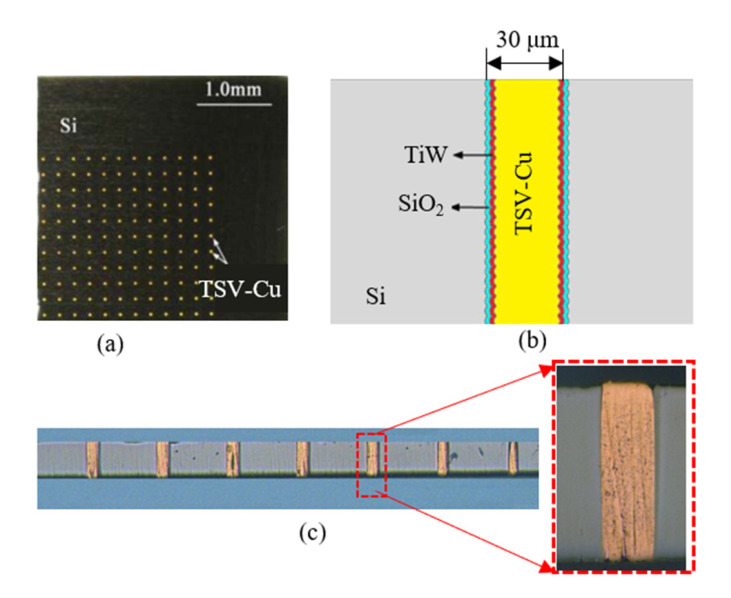
Schematic diagram of the TSV sample: (**a**) the TSV-Cu array; (**b**) cross-section of TSV-Cu; (**c**) polished axial cross-section of TSV-Cu.

The sample of the TSV arrays is shown in Figure 1a. The diameter, depth, and pitch distance of TSV-Cu are 30 μm, 100 μm, and 200 μm, respectively. The structure of the sample cross-section is shown in Figure 1b, including a TSV-Cu filled by electroplating, a TiW barrier layer deposited by physical vapor deposition (PVD), and a SiO_2_ insulation layer deposited by chemical vapor deposition (CVD). After that, three groups of samples were prepared. In the first group, the sample was fabricated at room temperature. In the second group, the sample was thermally cycled 10 times from 0 °C to 125 °C. The heating and cooling rates were both 10 °C/min, and the holding time at the two peak cycling temperatures was 2 min. In the third group, the sample was annealed at 250 °C. The annealing temperature was raised from room temperature to 250 °C, at a heating rate of 10 °C/min, then held for 30 min, followed by naturally cooling down to room temperature. Next, the three groups of samples were polished at the axial cross-section of TSV-Cu, with *#*2500 sandpaper, as shown in Figure 1c.

**Figure 2:**
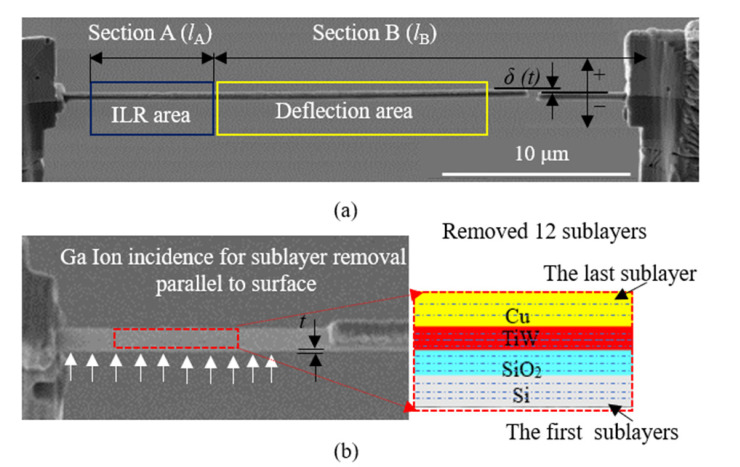
Schematic diagram of the micro cantilever beam sample: (**a**) SEM image of the micro cantilever beam for the Cu/TiW/SiO_2_/Si-interconnected structure; (**b**) SEM image of the removed sublayers.

As shown in Figure 2a, the micro cantilever beam sample of the TSV-Cu/TiW/SiO_2_/Si interconnected structure was fabricated via FIB. The detailed fabrication steps were given in the previous publication [35]. Scanning electron microscopy (SEM) (ZEISS, IGMA, Jena, Germany) was used to measure the lengths (*l*), widths (*w*), and thicknesses (*t*) of the three microcantilever beam samples, as well as the thicknesses of the interconnected materials, as shown in Table 1. The micro cantilever beam in Figure 2a is divided into two parts: Section A and Section B. The interconnected materials were removed layer by layer with FIB in the area of Section A. Figure 2b shows the SEM diagram of Section A after removing twelve sublayers of materials. The magnification area shows detailed materials of the twelve sublayers; the first sublayer is near the Si substrate, and the twelfth layer is TSV-Cu. Section B is referred to as the deflection area. The deflection *δ*(*t*) at the end of Section B, after removing every sublayer, was measured.

### 2.2. ILR Method

The main steps of measuring residual stress in TSV interconnected structure with ILR method are as follows:

(a) The initial deflection δ_origin_ was measured by SEM at the free end of the micro cantilever beam in Section B.
(b) The sublayers were removed, layer by layer, via FIB, from the top of the TSV-Cu to the bottom of the Si substrate in Section A. The thickness of each removed sublayer is in the nanoscale and the minimum thickness is 9 nm. With the decrease in thickness in Section A, the residual stress of the micro cantilever beam was constantly reconstructed, leading to deflection with different curvatures. The remaining thickness (*t*) in Section A and the corresponding deflection δ(*t*) in Section B were measured by SEM after removing each sublayer.
(c) The biaxial modulus (*E*_b_) of each sublayer was calculated according to *E*_b_ = *E*/(1 − *υ*), where *υ* is the Poisson’s ratio of the sublayer material. Given that *E*_Cu_ = 155.47 GPa, *E*_TiW_ = 129 GPa, *E*_SiO2_ = 72.1 GPa, and *E*_Si_ = 131 GPa.

The materials of each removed sublayer and the calculated biaxial modulus for the three micro cantilever beam samples are presented in Table 2, Table 3 and Table 4.

**Figure 3:**
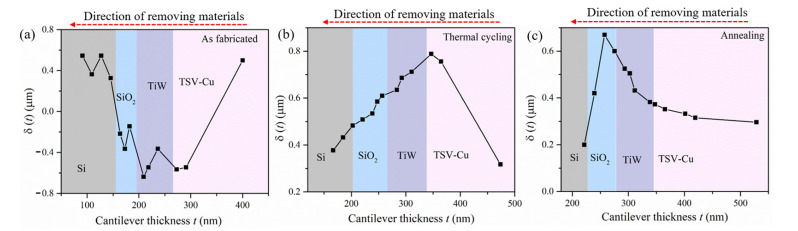
The deflection of samples, (**a**) as-fabricated; (**b**) thermal cycling; (**c**) annealing.

The deflection δ(*t*) of the three micro cantilever beam samples is shown in Figure 3. From Figure 3a, the deflection of the sample as fabricated changes from a positive value to a negative value with removing TSV-Cu. As the TiW and SiO_2_ are removed, the deflection fluctuates in the negative range, followed by increasing it to a positive value during the removal of Si. The deflection δ(*t*) of a thermally cycled sample is shown in Figure 3b; the deflection increases gradually during the removal of TSV-Cu, then decreases during the subsequent removal of materials. Figure 3c shows the δ(*t*) of the annealed sample; the deflection increases during the removal of TSV-Cu, TiW, and SiO_2_, then decreases with the removal of SiO_2_ and Si. It can be seen that deflection varies with the different thermal loading conditions.

## 3. Results and Analysis

### 3.1. Failure Modes of the TSV-Cu/TiW/SiO_2_/Si Interfaces

**Figure 4:**
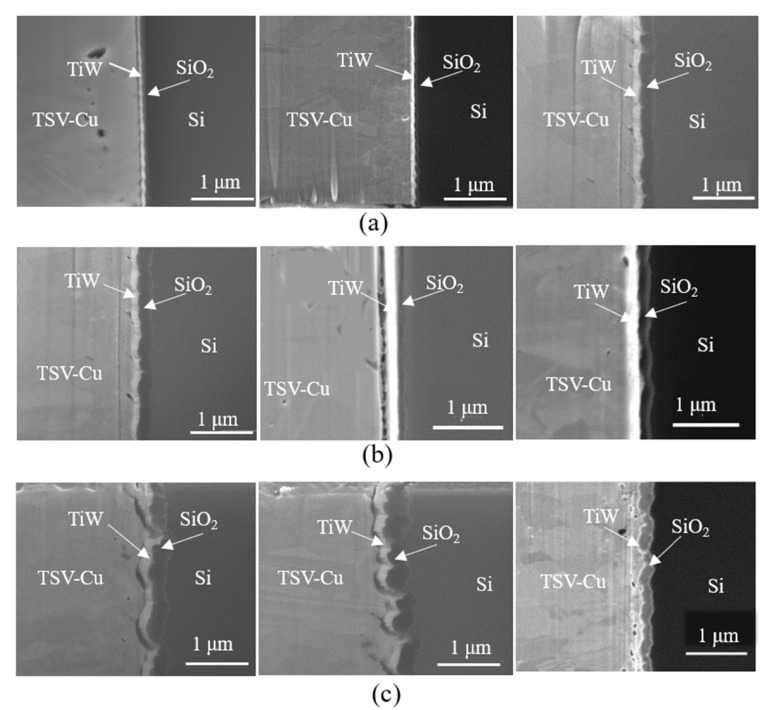
Failure modes of TSV-Cu/TiW/SiO_2_/Si interfaces: (**a**) as fabricated; (**b**) thermal cycling; (**c**) annealing.

The failure modes of the TSV-Cu/TiW/SiO_2_/Si interfaces for the three groups of samples were observed by SEM. Figure 4a displays the fact that many micro voids and cracks caused by the electroplating process form at the Cu/TiW interface, as fabricated. Figure 4b shows the interfaces of a thermally cycled sample; some TSV-Cu/TiW interfaces are intact, with a few micro voids, but penetrable cracks appear at the interfaces of TSV-Cu/TiW and TiW/SiO_2_, respectively. As shown from the annealed sample interfaces in Figure 4c, penetrative cracks occur at the interfaces of TSV-Cu/TiW and TiW/SiO_2_ synchronously, while some interfaces are still intact, with a number of micro voids. 

The above results indicate that the electroplating process would lead to the formation of initial micro voids and cracks at the TSV-Cu/TiW/SiO_2_/Si interfaces. After thermal cycling and annealing, micro voids would continue to form and coalesce; microcracks would then propagate to form penetrative cracks. These failure phenomena are presumed to be related closely with the stress concentration and microstructure evolution at the interface, under different thermal loadings.

### 3.2. Residual Stress Distribution

**Figure 5:**
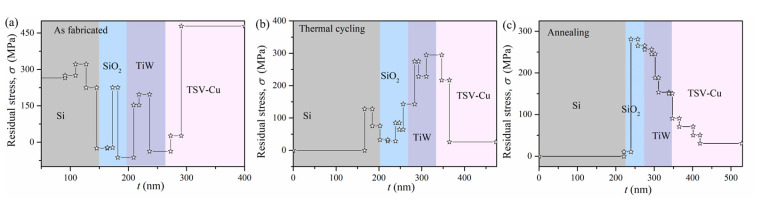
The residual stress distributions of each sublayer in the three samples under different thermal loading conditions: (**a**) as fabricated; (**b**) thermal cycling; (**c**) annealing.

The residual stress distributions under different thermal loading conditions are shown in Figure 5a–c. It is found that the residual stress at both the interfaces and in the inner individual materials exhibit dramatic fluctuations.

**Figure 6:**
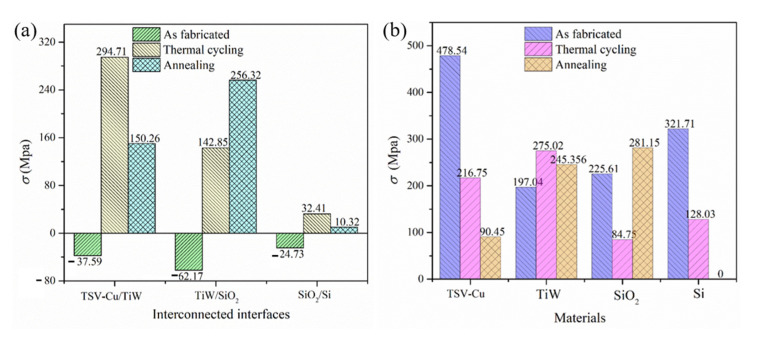
The residual stress at interfaces and inner materials: (**a**) residual stress at interfaces; (**b**) residual stress in inner materials.

The residual stresses at the interfaces of TSV-Cu/TiW, TiW/SiO_2_, and SiO_2_/Si are displayed in Figure 6a. The residual stress at the interfaces of TSV-Cu/TiW, TiW/SiO_2_, and SiO_2_/Si changes from small compressive stress at room temperature to large tensile stress after thermal cycling. Specifically, the residual stress at the TSV-Cu/TiW interface changes from −37.59 MPa to 294.71 GPa, while the residual stress at the TiW/SiO_2_ interface changes from −62.17 MPa to 142.85 MPa, and the residual stress at the SiO_2_/Si interface changes from −24.73 MPa to 32.41 MPa. After annealing, the residual stresses at the interfaces of TiW/SiO_2_, TiW/SiO_2,_ and SiO_2_/Si are 150.26 GPa, 256.32 MPa, and 10.32 MPa, respectively. It is worth noting that both thermal cycling and annealing cause the interfacial compressive stress to become tensile stress. Furthermore, according to the magnitude of these interfacial residual stresses, the condition of annealing seems to be beneficial to the interfaces of TSV-Cu/TiW and SiO_2_/Si, but the condition of thermal cycling is better for TiW/SiO_2_ interface. It can be speculated that tensile stress is one of the most important mechanisms for the generation of interface micro cracks.

As for the TSV-Cu/TiW and TiW/SiO_2_ interfaces, the residual stress is within the range of 140~300 MPa after thermal cycling or annealing, which is at a considerable level. The main reason for this is the larger CTE mismatch for TSV-Cu (22.8 × 10^−6^), TiW (6.2 × 10^−6^), and SiO_2_ (1 × 10^−6^) [21,36]. The CTE of TSV-Cu is three times higher than that of TiW. The CTE of TiW is also nearly six times that of SiO_2_. However, as for the SiO_2_/Si interface, the residual stress is within 30 MPa, which is at a lower level. It is because the CTEs between SiO_2_ and Si are smaller and show little difference. Furthermore, other parameters, such as the elastic modulus and the generated intermetallic compounds at these interfaces, have also influenced the residual stresses at various material interfaces. For example, the Ti and Cu atoms would generate Ti-Cu intermetallic compounds at the TSV-Cu/TiW interface under high temperatures [37,38]. The Ti, O, and Si atoms would also form titanium oxide and titanium silicon compounds at the TiW/SiO_2_ interface [39,40]. These intermetallic compounds can enhance the interface strength and can affect the residual stress at the TSV-Cu/TiW interface.

The maximum residual stresses of the inner individual materials are shown in Figure 6b. The residual stress of the inner TSV-Cu decreases from 478.54 MPa to 216.75 MPa after thermal cycling and reduces to 90.45 MPa after annealing. The residual stress of the inner TiW after thermal cycling and annealing are 275.02 MPa and 245.36 MPa, respectively; both of them are higher than that of 197.04 MPa, as fabricated. The residual stress inner SiO_2_ is 84.75 MPa, under thermal cycling, which is less than the stress as fabricated at 225.61 MPa and the annealed stress at 281.15 MPa. The residual stress of inner Si, as fabricated, is 320 MPa, which is similar to the stress measured by the nanoindentation test in [17]. The residual stress in Si is mainly influenced by the stress in TSV-Cu. Moreover, the residual stress as high as 100 MPa in Si would cause a 7% shift in carrier mobility of the device transistor in Si [12]. The stress-influence region on the device transistor in Si is defined as the keep-out-of-zone (KOZ), where the placement of devices is avoided. Besides this, the residual stress in Si decreased with the increase in the distance to the TSV-Cu edge [41]. Therefore, after thermal cycling, the residual tensile stress in Si is 128 MPa, as the distance is very close to the TSV-Cu edge; then, the residual stress decreases to zero as the distance is far away from the TSV-Cu edge. A similar variation tendency of residual stress in Si was observed in [12]. After annealing, the residual stress in Si is nearly zero. Investigations showed that the residual stresses in Si for the systems of Si/SiOx/Ni and Si/TiN/W were also zero, measured with the ILR method [27,29]. The reason is that the residual stress in Si after annealing is nearly reduced to zero, and the removed thickness of the Si material is relatively large, resulting in a zero average-thickness residual stress in the Si, based on the ILR method. 

### 3.3. Microstructure Analysis

**Figure 7:**
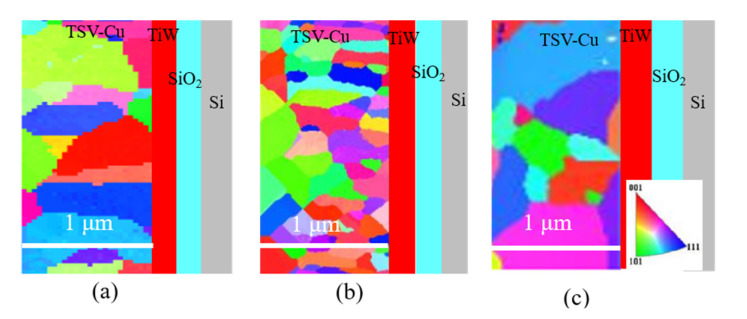
EBSD images of TSV-Cu under the different thermal loading conditions: (**a**) as fabricated; (**b**) thermal cycling; (**c**) annealing.

The variations of the stress at interfaces and the inner material might be related to the microstructure evolution at high temperatures. Figure 7 shows the EBSD images of TSV-Cu at the TSV-Cu/TiW interface, under different thermal loading conditions. The TSV-Cu grains are formed in irregular polygonal shapes, with random orientation.

**Figure 8:**
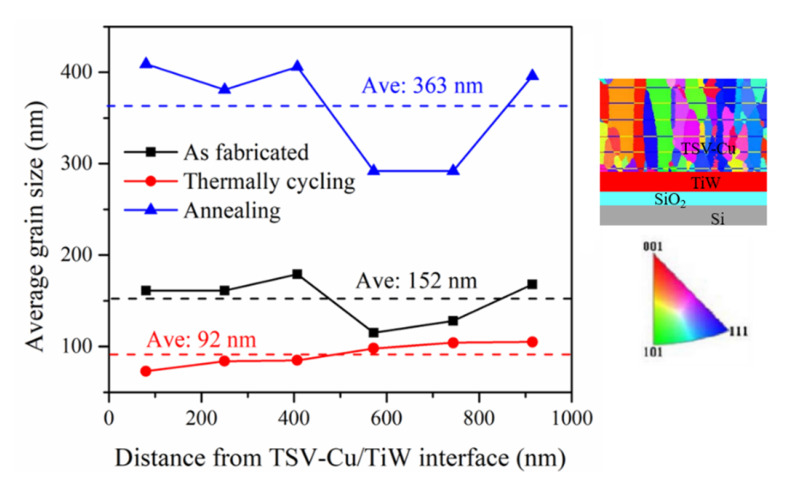
The distribution of TSV-Cu grain size according to distance from the TSV-Cu/TiW interface.

Figure 8 shows the TSV-Cu grain sizes under different thermal loading conditions. The average grain size of TSV-Cu as fabricated is 152 nm and decreases to 92 nm after thermal cycling; however, it increases to 363 nm after annealing. The grain size distribution in the perpendicular direction to the TSV-Cu/TiW interface was evaluated with the linear intercept method. The pitch distance of the intercept line was 160 nm. It can be seen that the distribution of the TSV-Cu grain size is relatively stable under thermal cycling, while the distributions of the annealed grain size or the grain size as fabricated exhibit sharp fluctuations.

**Figure 9:**
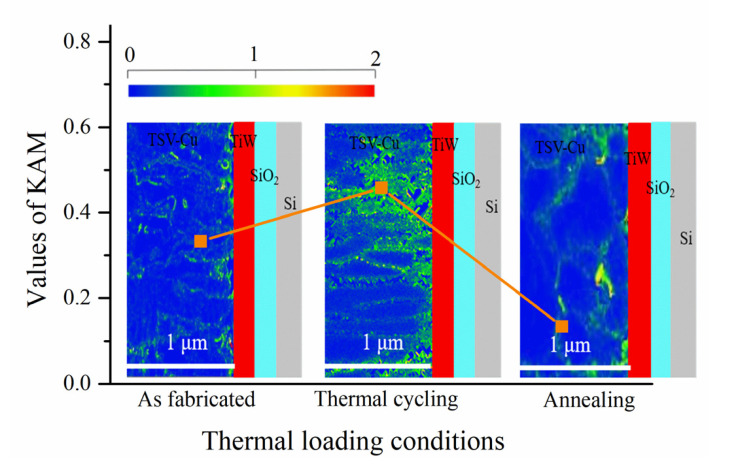
The local misorientation distribution of TSV-Cu under different thermal loading conditions.

The local misorientation could reflect the level of dislocation density and micro strain. Figure 9 shows the local misorientation under different thermal loading conditions. The higher strain is concentrated on the grain boundaries or specific inner individual grains. As shown in Figure 9, the kernel average misorientation (KAM) value of the sample as fabricated is 0.333. After thermal cycling, the KAM value increases to 0.458. Moreover, the KAM value of the TSV-Cu decreases to 0.134 after annealing. It can be inferred that the different residual stress levels under thermal loadings motivate two different microstructure evolution mechanisms of TSV-Cu.

### 3.4. Influence of the TSV-Cu Microstructure on Failure Modes and Residual Stress

**Figure 10:**
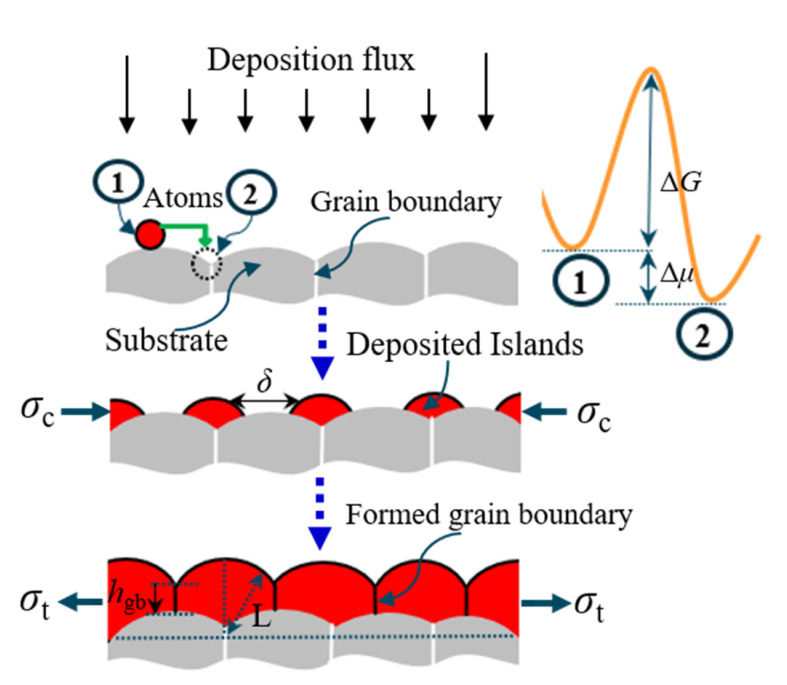
Formation mechanism of the residual stress during the deposition process.

It is worth mentioning that the variation trends of residual stresses at the interfaces of Si/SiO_2_, SiO_2_/TiW, and TiW/TSV-Cu are similar for the sample as fabricated. In other words, the residual stress is compressive within tens of nanometers at these interfaces and became tensile with the increase in material thickness. The formation mechanism of residual stress as fabricated can be traced back to the deposition process shown in Figure 10. During the deposition process, the red atoms of the deposited material diffuse from the free surface site ① of the substrate material to the grain boundary site ②. Compressive stress forms in the grain boundary, due to the difference in chemical potential energy at the sites of ① and ② [42]. In addition, *N*_i_ atoms diffuse to the grain boundary to form deposited islands; the resulting compressive stress can be expressed using Equation (1), as follows:

(1) $$\sigma_{c}=M_{f}\frac{N_{i}\cdot a}{L}$$

where *M*_f_ is the biaxial modulus of the substrate, *a* is the diameter of the deposited atoms, and *L* is the radius of the formed islands. *N*_i_ is related to the deposition process; the number of atoms per unit time can be presented by Equation (2):

(2) $$\frac{\partial N_{i}}{\partial t}{=4C}_{s}\frac{D}{a^{2}}(1-e^{\frac{-\Delta \mu }{kT}})$$

where C_s_ is related to the material property. *D* is the effective diffusivity, representing the ratio of surface diffusivity to the grain boundary diffusivity. Δ*μ* is the difference in chemical potential energy between the surface and the grain boundary. *K* is the gas constant and *T* is the deposition temperature. With an increase in the thickness of the deposited material, compressive stress turns to tensile stress, as shown in Figure 5a. The changing of the residual stress is related to the coalescence of the islands. During the subsequent deposition, the islands continue their growth, reducing their pitch distance, *δ*. Finally, the islands gather to form a continuous deposited film. Thus, the free surface of the islands is transformed into grain boundaries, resulting in a decrease in the total interfacial energy. The reduced interfacial energy ($\Delta E_{\mathrm{interface}}$) is expressed as in Equation (3). The gathering of islands induces a uniform biaxial strain between the islands, which raises the strain energy ($\Delta E_{\mathrm{strain}}$), as expressed in Equation (4). The new grain boundaries are generated if $\left|\Delta E_{\mathrm{interface}}\right|$ is greater than $\left|\Delta E_{\mathrm{strain}}\right|$. Meanwhile, the critical tensile stress ($\sigma_{crit}$) forms between the islands, and can be expressed as in Equation (5):

(3) $$\Delta E_{\mathrm{interface}}=-4Lh_{gb}(\gamma_{sur}-\frac{1}{2}\gamma_{gb})$$

(4) $$\Delta E_{\mathrm{strain}}=Lh_{gb}^{2}\frac{(1-v)\sigma_{t}}{E}$$

(5) $$\sigma_{crit}=2{\left(\frac{E}{1-v}\right)}^{1/2}{\left(\frac{\Delta \gamma }{L}\right)}^{1/2}$$

where *h*_gb_ is the height of the new grain boundary, $\gamma_{sur}$ is the surface energy, and $\gamma_{gb}$ is the grain boundary energy. *E* and *v* are the elastic modulus and Poisson’s ratio, respectively. The stress variations were also observed at W/TiN and Si/TiN interfaces [30,43]. Nevertheless, the residual stress at the initial interface displayed a tensile state if the depth resolution exceeded tens of nanometers [27]. Furthermore, the tensile stress also occurred at the interface within tens of nanometers at a higher deposition rate [44]. This was because the high deposition rate resulted in the rapid formation of denser islands with a high aspect ratio. These islands frequently coalesced with each other to form new grain boundaries, leading to the generation of tensile stress during the initial deposition stage.

During thermal cycling, tensile and compressive stress are constantly generated at the interconnected interfaces due to the significant difference in CTE between TSV-Cu and Si. Moreover, the stress would accumulate continuously owing to the anisotropy of the elastic parameters and the initial micro defects of each grain. Thus, the KAM value shown in Figure 9 increases, as well as the internal strain energy of the grains, reaching that of the energy of recrystallization. Recrystallization promotes nucleation, resulting in a smaller grain size, as shown in Figure 8. According to Equation (5), the small grain size indicates the increase in tensile stress, which leads to variations in the residual stress at the TSV-Cu/TiW interface. Lei et al. [45] also observed that the fraction of the TSV-Cu recrystallization increased after thermal cycling at low temperatures. The residual stress of the inner TSV-Cu after thermal cycling is lower than that as fabricated, due to the stress relief induced by recrystallization.

After annealing, the TSV-Cu grain size increases significantly; dense micro voids and cracks form at the interfaces, resulting in a decrease in residual stress at both interfaces and the inner TSV-Cu, according to Equation (5).

## 4. Discussion

### 4.1. Numerical Procedure of the ILR Method

**Figure 11:**
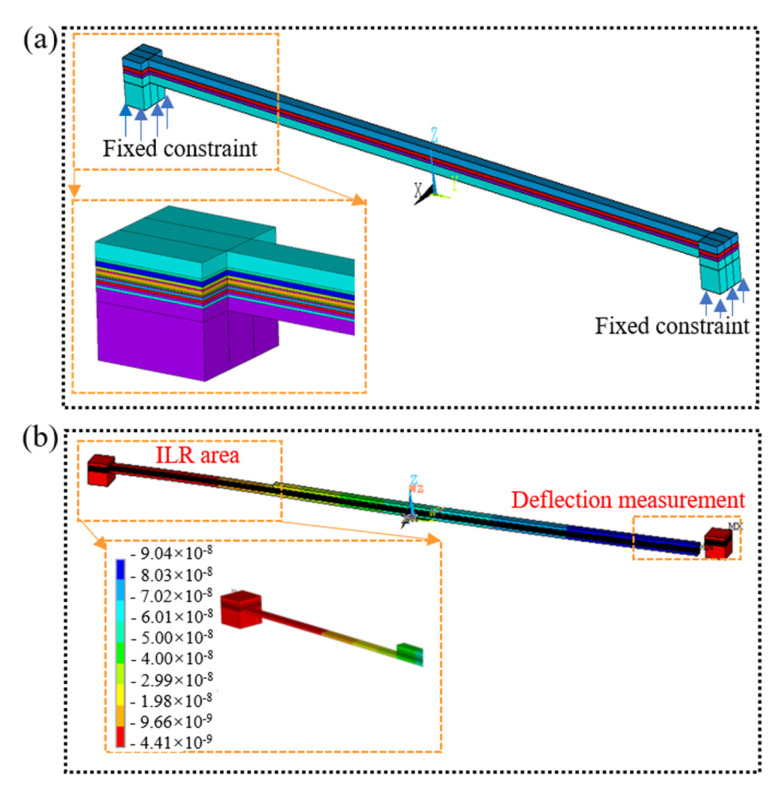
FE model and deflection contour: (**a**) FE model of the micro cantilever beam; (**b**) deflection contour after removing the last sublayer.

**Figure 12:**
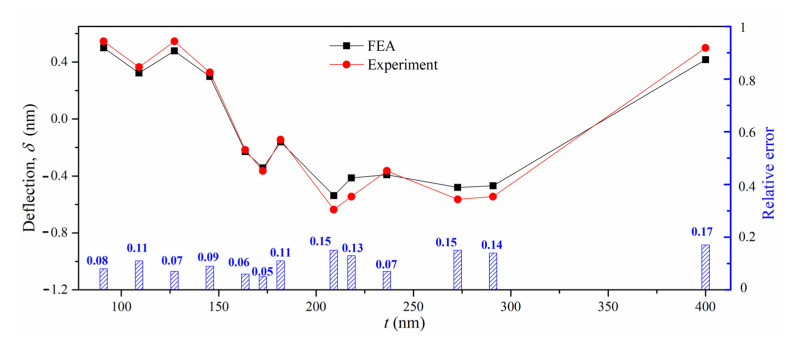
The comparison of the measured deflection and simulated deflection.

The sublayer-removing processes of the micro cantilever beam sample were simulated by FEM to verify the applicability of the ILR method. As shown in Figure 11a, the FE model of the micro cantilever beam was built based on ANSYS software (2019); the two ends of the cantilever beam were fixed and hexahedral elements with the numbers of 135,760 were adopted. The model was divided into 13 sublayers, and the measured residual stress of sublayers for the sample as fabricated was taken as the loading to apply in the FE model. The detailed material parameters of each sublayer are given in Table 2. The elemental birth and death technologies were utilized to simulate the removal of each sublayer. The residual stress in the micro cantilever beam was rebalanced after removing each sublayer. The contour of deflection after removing the last sublayer is shown in Figure 11b. The simulated deflection *δ*_FEA_ is compared with the measured deflection, *δ*_Exp_, and the relative error formula is expressed as $\left|\frac{\delta_{\mathrm{FEA}}-\delta_{\mathrm{Exp}}}{\delta_{\mathrm{Exp}}}\right|$. The comparison is shown in Figure 12. The measured deflection and the simulated deflection are highly coincident, and the relative error is 0.05~0.17. The results verify the applicability of the ILR method to measure the nanoscale depth profiles for the stress gradient of the TSV Cu/TiW/SiO_2_/Si multilayer interface.

### 4.2. Influence of the Geometric Error of the Micro Cantilever Beam

**Figure 13:**
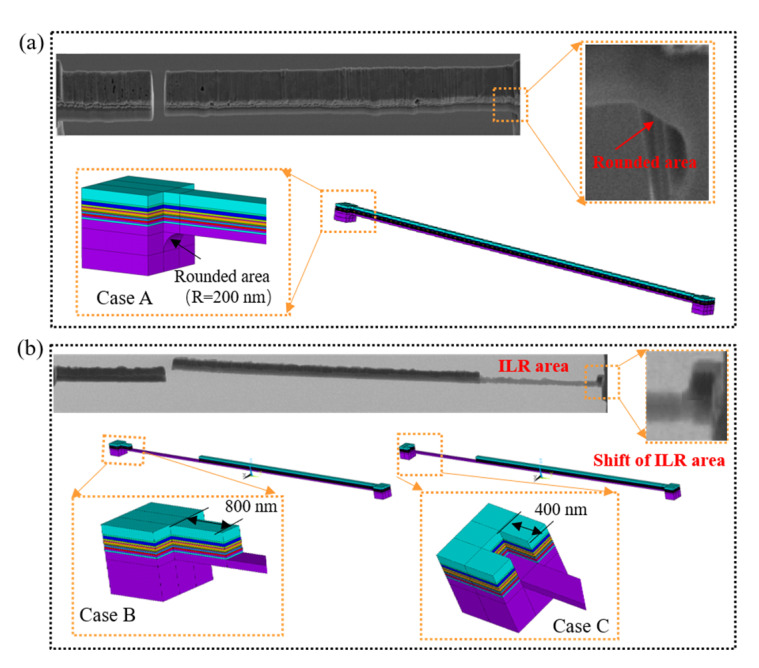
The SEM images and FE models of the micro cantilever beam with three kinds of machine errors: (**a**) rounded area; (**b**) shifts in the ILR area.

**Figure 14:**
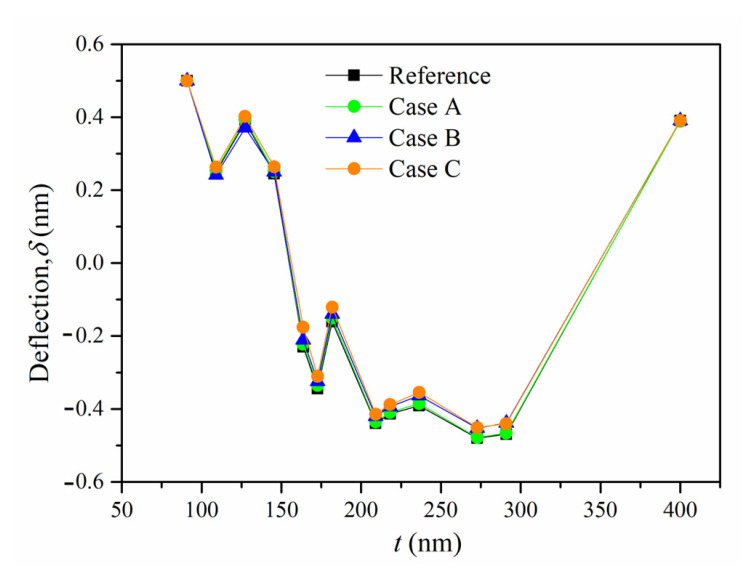
The deflection comparisons between the reference model and the three cases with a geometric error.

It is worth noting that the geometrical dimensions of the micro cantilever beam fabricated by FIB are different from the ideal dimensions. For example, the SEM image in Figure 13a shows that a rounded area is formed at the root of the micro cantilever beam, rather than an ideal square edge. In addition, the SEM diagram in Figure 13b shows that the ILR areas have some shifts, i.e., the areas might shift forward or backward. Herein, three FE models with the above geometric errors, i.e., Case A, Case B, and Case C, are constructed to investigate their influences on the measured results. The radius (R) of the rounded area is amplified to be 200 nm for Case A. As for Case B and Case C, the shift distances of the ILR area are magnified to be forward 800 nm and backward 400 nm, respectively. Moreover, the measured residual stresses of the fabricated sample are treated as mechanical loadings to apply to the three models. The simulated deflections of the three cases are recorded after removing each sublayer. Besides this, the simulated deflections are compared with that of the reference model with ideal dimensions. The relative error formula is expressed as $\left|\frac{\delta_{\mathrm{Case}}-\delta_{\mathrm{Ref}}}{\delta_{\mathrm{Ref}}}\right|$. The results are shown in Figure 14. The deflections of Case A are reduced slightly, owing to the increased stiffness caused by the rounded area. Additionally, the relative errors between Case A and the reference model are within 0.05, which indicates negligible effects on the measured results. Furthermore, the relative errors between Case B, Case C, and the reference model are less than 0.03, which also leads to ignorable effects on the measured results. Nevertheless, since the actual geometrical errors of the micro cantilever beams in the experiment did not reach the magnitudes of Case A, Case B, and Case C, their influences on the measured results could basically be ignored.

### 4.3. Influence of Material Damage

**Figure 15:**
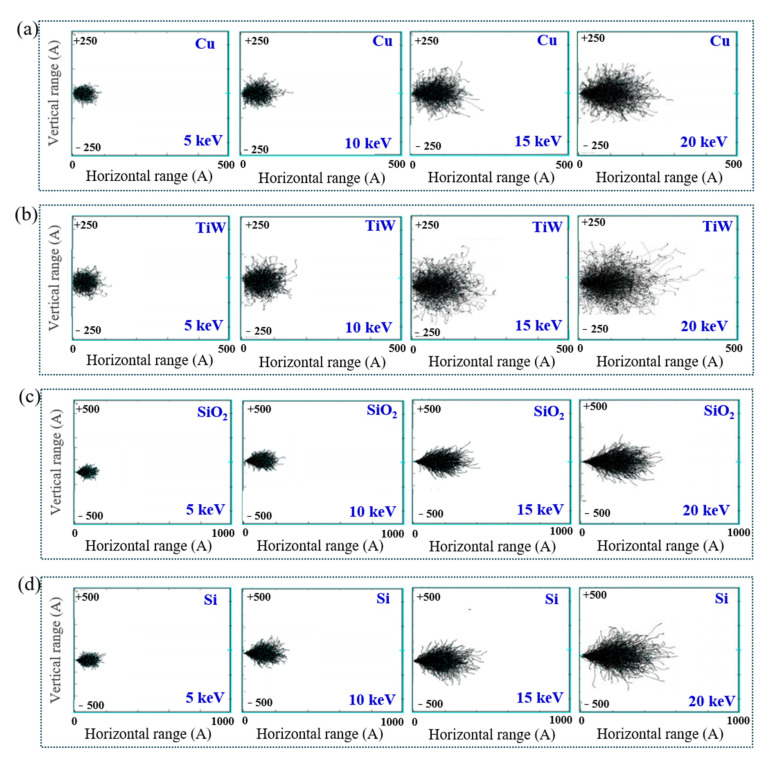
Incidence trajectories of Ga^+^ in various materials with different energies: (**a**) Cu; (**b**) TiW; (**c**) SiO_2_; (**d**) Si.

The Ga^+^ beam was utilized by FIB to machine the TSV interconnected structure for preparing the micro cantilever beam samples. However, the Ga^+^ beam might cause initial damage to the interconnected materials, affecting the accuracy when measuring the residual stress gradient. Herein, the influence of kinetic energy for Ga^+^ beam grazing incidence in terms of material damage is explored with the SRIM software (2013) [46]. One thousand Ga^+^ are incident horizontally to the materials of TSV-Cu, TiW, SiO_2_, and Si in the SRIM program. The incident angle was set at 0 degrees, and the kinetic energies varied from 5 keV to 30 keV. The incidence trajectories of Ga^+^ for different materials were analyzed. Figure 15 shows the trajectory diagrams of the Ga^+^ beam incident on each material with different energies. The horizontal ranges of Ga^+^ in materials of Cu, TiW, SiO_2_, and Si exhibit increasing trends with the increase of energies. Furthermore, the incident trajectories for different materials have transverse deviations in terms of vertical range, and the transverse deviation ranges also display increasing trends with the increase in energies. Nevertheless, the transverse deviation amplitude would directly affect the measurement accuracy of the residual stress.

**Figure 16:**
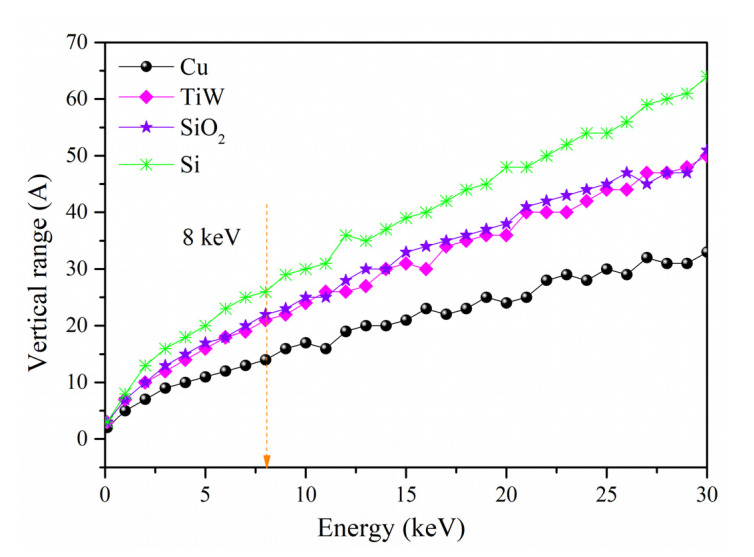
The maximum transverse deviations of Ga^+^ in various materials with different energies.

Transverse deviations in the TSV-Cu/TiW/SiO_2_/Si interconnected materials with different energies are shown in Figure 16. The maximum transverse distances at the energy of 30 keV are 3.4 nm in Cu, 5.0 nm in TiW, 5.1 nm in SiO_2_, and 6.3 nm in Si, respectively. The above transverse deviations would induce significant material damage, resulting in large experimental errors since the minimum thickness of the removal sublayer is only 9 nm. Nevertheless, the actual energy of the Ga^+^ beam adopted in the experiment is 8 keV, and the simulated maximum transverse deviations in Cu, TiW, SiO_2_, and Si are 1.6 nm, 2.2 nm, 2.3 nm, and 2.9 nm, respectively. The introduced material damage is negligible, which indicates that the energy parameter of the Ga^+^ beam used in this study is reasonable.

## 5. Conclusions

The residual stress of a TSV-Cu/TiW/SiO_2_/Si interconnected structure under different thermal loadings was measured using the ILR method. The failure modes of the interconnected interfaces were observed. The influence mechanisms of microstructure evolution on the residual stress gradient were analyzed. In addition, the accuracy of the ILR method was analyzed using FEM. The following conclusions can be drawn:

1. The residual stresses at the as-fabricated interfaces of TSV-Cu/TiW, TiW/SiO_2_, and SiO_2_/Si were −37.59 MPa, −62.17 MPa, and −24.73 MPa, respectively. After thermal cycling or annealing, the residual stresses at the interfaces of TSV-Cu/TiW and TiW/SiO_2_ ranged from 140 MPa to 300 MPa. However, the residual stress at the interfaces of SiO_2_/Si was within the range of 10~40 MPa.
2. The maximum residual stress inside the fabricated TSV-Cu was 478.54 MPa; it decreased to 216.75 MPa after thermal cycling and was reduced to 90.45 MPa after annealing. The maximum residual stress in the inner TiW was at a considerable level, ranging from 190 MPa to 280 MPa under various thermal loadings. The maximum residual stress of the inner SiO_2_ was 84.75 MPa after thermal cycling, which was the smallest value compared with that under other thermal conditions. The maximum residual stress in the inner fabricated Si was 321.71 MPa, which decreased to 128 MPa and zero after thermal cycling and annealing, respectively.
3. The initial micro voids and cracks at the interfaces of the TSV-Cu/TiW/SiO_2_/Si were caused by the electroplating process. The micro voids became dense and the micro-cracks propagated to form penetrating cracks after thermal cycling and annealing. In addition, the average grain size of the TSV-Cu was 152 nm as fabricated, then it decreased to 92 nm and was distributed uniformly around the TSV-Cu/TiW interface after thermal cycling. Furthermore, the average grain size enlarged to 363 nm and exhibited fluctuant distribution around the TSV-Cu/TiW interface after annealing.
4. Residual stresses were generated and accumulated at the interconnected interfaces during thermal cycling, which increased the KAM value and motivated the recrystallization nucleation of TSV-Cu grains. Thus, many new grain boundaries formed, resulting in a decrease in the grain size and an increase in tensile stress at the TSV-Cu/TiW interface. During annealing, the high annealing temperature and dense micro voids released the residual stress of the inner TSV-Cu, which induced a decrease in the KAM value and the overall stress in the inner TSV-Cu.
5. The applicability of the ILR method to measure the nanoscale residual stress gradient of the TSV Cu/TiW/SiO_2_/Si multilayer interfaces was verified by FEM. It was also confirmed that the geometrical errors of the micro cantilever beam and the adopted Ga^+^ kinetic energy of FIB were reasonable.

## Figures and Tables

**Table 1:** Dimensions of the micro cantilever beam.

	Length, *l* (nm)	Width, *w* (nm)	Thickness, *t* (nm)	TSV-Cu (nm)	TiW (nm)	SiO_2_ (nm)	Si (nm)
As fabricated	23,630	270	400	130	70	50	150
Thermal cycling	24,880	271	474	137	73	59	205
Annealing	23,970	220	528	206	65	52	225

**Table 2:** As-fabricated samples.

Sublayer, *i*	0–3	4	5–6	7	8–9	10	11–12
Material	Si	Si/SiO_2_	SiO_2_	SiO_2_/TiW	TiW	TiW/Cu	Cu
*E_b_*_–*i*_/GPa	201.54	119.42	86.87	138.36	215.00	216.63	222.10

**Table 3:** Samples after thermal cycling.

Sublayer, *i*	0–2	3	4–6	7	8–9	10	11–12
Material	Si	Si/SiO_2_	SiO_2_	SiO_2_/TiW	TiW	TiW/Cu	Cu
*E_b_*_–*i*_/GPa	201.54	109.63	86.87	183.71	215.00	219.94	222.10

**Table 4:** Samples after annealing.

Sublayer, *i*	0	1	2–3	4	5–7	8	9–12
Material	Si	Si/SiO_2_	SiO_2_	SiO_2_/TiW	TiW	TiW/Cu	Cu
*E_b_*_–*i*_/GPa	201.54	97.56	86.87	200.45	215.00	218.53	222.10

## Data Availability

The data will be available on reasonable request.
